# Extensive Cortical Convergence to Primate Reticulospinal Pathways

**DOI:** 10.1523/JNEUROSCI.1379-20.2020

**Published:** 2021-02-03

**Authors:** Karen M. Fisher, Boubker Zaaimi, Steve A. Edgley, Stuart N. Baker

**Affiliations:** ^1^Medical School, Newcastle University, Newcastle upon Tyne NE2 4HH, United Kingdom; ^2^School of Life and Health Sciences, Aston University, Birmingham B4 7ET, United Kingdom; ^3^Department of Physiology, Development and Neuroscience, Cambridge University, Cambridge CB2 3DY, United Kingdom

**Keywords:** intracellular, primate, reticular formation

## Abstract

Early evolution of the motor cortex included development of connections to brainstem reticulospinal neurons; these projections persist in primates. In this study, we examined the organization of corticoreticular connections in five macaque monkeys (one male) using both intracellular and extracellular recordings from reticular formation neurons, including identified reticulospinal cells. Synaptic responses to stimulation of different parts of primary motor cortex (M1) and supplementary motor area (SMA) bilaterally were assessed. Widespread short latency excitation, compatible with monosynaptic transmission over fast-conducting pathways, was observed, as well as longer latency responses likely reflecting a mixture of slower monosynaptic and oligosynaptic pathways. There was a high degree of convergence: 56% of reticulospinal cells with input from M1 received projections from M1 in both hemispheres; for SMA, the equivalent figure was even higher (70%). Of reticulospinal neurons with input from the cortex, 78% received projections from both M1 and SMA (regardless of hemisphere); 83% of reticulospinal cells with input from M1 received projections from more than one of the tested M1 sites. This convergence at the single cell level allows reticulospinal neurons to integrate information from across the motor areas of the cortex, taking account of the bilateral motor context. Reticulospinal connections are known to strengthen following damage to the corticospinal tract, such as after stroke, partially contributing to functional recovery. Extensive corticoreticular convergence provides redundancy of control, which may allow the cortex to continue to exploit this descending pathway even after damage to one area.

**SIGNIFICANCE STATEMENT** The reticulospinal tract (RST) provides a parallel pathway for motor control in primates, alongside the more sophisticated corticospinal system. We found extensive convergent inputs to primate reticulospinal cells from primary and supplementary motor cortex bilaterally. These redundant connections could maintain transmission of voluntary commands to the spinal cord after damage (e.g., after stroke or spinal cord injury), possibly assisting recovery of function.

## Introduction

Multiple descending pathways transmit motor commands to the spinal cord. In mammals the corticospinal tract has become the dominant system, especially in primates, where powerful corticospinal connections underlie fine dexterous abilities (Lemon, 2008). The reticulospinal tract (RST) is a major parallel system involved in posture and gross motor function (Peterson et al., 1975, 1978; Iwamoto and Sasaki, 1990; Iwamoto et al., 1990; Isa and Sasaki, 2002; Drew et al., 2004; Schepens and Drew, 2004), although it also contributes to upper limb function, even to fine hand control (Davidson and Buford, 2006; Riddle et al., 2009; Riddle and Baker, 2010; Soteropoulos et al., 2012).

Reticulospinal neurons originate throughout the pontomedullary reticular formation (Sakai et al., 2009), which receives converging sensory inputs from visual, auditory, cutaneous, proprioceptive, and vestibular systems (Peterson and Abzug, 1975; Grantyn and Grantyn, 1982; Irvine and Jackson, 1983; Drew et al., 1996; Leiras et al., 2010). There are also inputs from cortical motor regions, allowing reticulospinal transmission of voluntary commands. Corticoreticular projections include collaterals of corticospinal neurons (Keizer and Kuypers, 1989); in the cat, some corticofugal fibers connect directly to reticulospinal neurons (He and Wu, 1985).

Keizer and Kuypers (1984, 1989) showed that corticoreticular projections arise from both contralateral motor and premotor cortex (PM) in cat and monkey. Matsuyama and Drew (1997) and Rho et al. (1997) extended this to show that inputs arose bilaterally, and were especially strong from the forelimb cortical representation. More recently, Fregosi et al. (2017) investigated primate corticoreticular projections using anterograde tracers injected in primary motor cortex (M1), the supplementary motor area (SMA) and the lateral PM. All regions made bilateral projections to both pontine and medullary nuclei of the reticular formation; however, there were generally more projections ipsilaterally for SMA and PM, and contralaterally for M1. In agreement, Darling et al. (2018) assessed projections from SMA to the medullary reticular formation; bouton numbers were very similar ipsilaterally and contralaterally.

Electrophysiological analysis of corticoreticular projections in cats reveals that many are collaterals of fast corticospinal neurons, although there is also a dedicated corticoreticular system without corticospinal collaterals (Jinnai, 1984; Lamas et al., 1994; Kably and Drew, 1998). Intracellular recordings from reticulospinal cells in cats reveals inputs from multiple cortical areas bilaterally (He and Wu, 1985). In monkey, transcranial magnetic stimulation over both ipsilateral and contralateral motor cortex excites reticular formation neurons at latencies compatible with corticoreticular pathways (Fisher et al., 2012).

Following corticospinal damage, reticulospinal connections strengthen (Zaaimi et al., 2012) and cell activity in the reticular formation alters (Zaaimi et al., 2018b), which may contribute to recovery (Xu et al., 2017). Extensive cortical lesions involving both M1 and S1 lead to an increase in corticoreticular connections from the intact ipsilesional SMA to the gigantocellular reticular formation (Darling et al., 2018); this correlates with measures of hand functional recovery. By contrast, Fregosi et al. (2018) showed there were fewer corticoreticular connections from the PM after lesion of M1, and from both PM and M1 in a monkey model of Parkinson's disease. McPherson et al. (2018) and Wilkins et al. (2020) demonstrated that an increased reliance on ipsilateral cortico-reticulospinal circuits following stroke may be responsible for the damaging flexor synergy sometimes seen. Choudhury et al. (2019) showed that stroke survivors with increased reticulospinal output had worse hand function. Reconciling these conflicting views of the positive versus negative contribution of the cortico-reticulospinal system to recovery will likely require a better understanding of the different cortical and reticular components of the system, and the laterality of projections. The primate corticospinal tract has developed new connections compared with other mammals (Lemon and Griffiths, 2005; Isa et al., 2007), and the PM comprises multiple specialized areas (Rizzolatti et al., 1998). To have the greatest relevance to patients, it is important to understand corticoreticular connections in a primate species closely similar to humans.

Here, we characterized corticoreticular inputs to the primate nucleus gigantocellularis of the medulla using both intracellular and extracellular recordings. We reveal extensive convergence from both hemispheres, and from different motor cortical areas, to single reticulospinal neurons.

## Materials and Methods

All animal procedures were conducted under United Kingdom Home Office regulations in accordance with the Animals in Scientific Procedures Act, 1986, and were approved by the Local Research Ethics Committee of Newcastle University. Recordings were made from five terminally anaesthetized adult rhesus macaque monkeys (*Macaca mulatta*; four females: monkeys S, 7.5 kg; J, 7 kg; A, 6.2 kg; Sh, 7.5 kg; one male: monkey R, 10.8 kg).

### 

#### Surgical preparation

All procedures were performed in a non-recovery setting. Surgery was performed under deep general anesthesia maintained with inhaled sevofluorane (2–4.5% in 100% O_2_) and supplemented with continuous intravenous infusion of alfentanil (8–21 µg kg^−1^ h^−1^). Initial preparation included a tracheotomy, and insertion of central lines via the carotid artery and external jugular vein. The bladder was catheterized to allow urine drainage. Hartmann's solution was infused to prevent dehydration (5–10 ml kg^−1^ h^−1^ including drug solutions). Methylprednisolone was given to reduce edema (initial loading dose of 30 mg kg^−1^, followed by 5.4 mg kg^−1^ h^−1^). Antibiotics were given to prevent sepsis (cefotaxime 250 mg, every 12 h).

**Figure 1.**
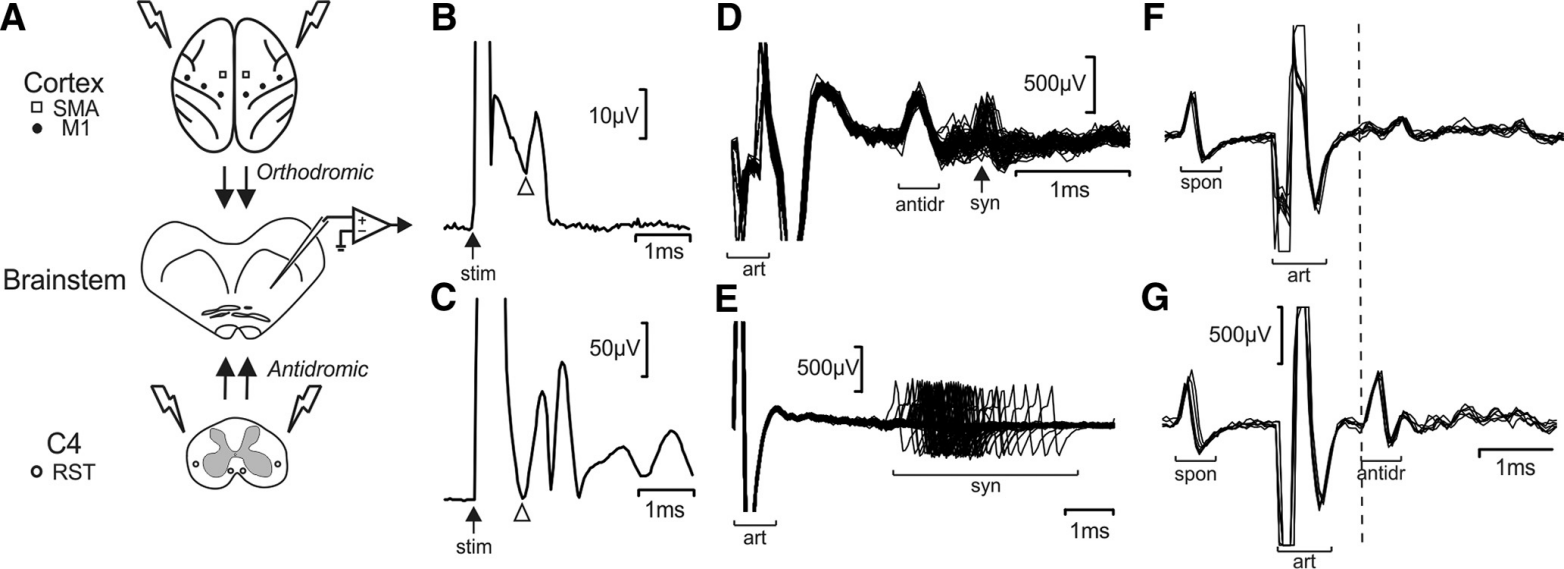
Recording methods. ***A***, Schematic showing arrangement of the experiment. Recordings were made from the reticular formation in the brainstem, using either glass micropipette (intracellular recordings) or metal electrodes (extracellular). Stimulation at the spinal cord lateral funiculi (C4 segment) allowed antidromic activation of reticulospinal neurons. Surface stimulation of the cortex over the M1 and SMA bilaterally activated corticoreticular projections. ***B***, Example field potential recordings from the brainstem surface made simultaneously with intracellular single unit recordings, to allow measurement of the corticofugal axon volley latency (arrow). ***C***, As ***B*** but made simultaneously with extracellular recordings. ***D***, ***E***, Example overlain sweeps from extracellular recordings illustrating the difference between antidromic spikes (antidr) with a fixed latency, compared with the high jitter observed in synaptically-evoked spikes (syn) following the stimulus artifact (art). ***F***, ***G***, Collision test. A spontaneous spike (spon) triggered spinal stimulation, which produced a stimulus artifact (art). An antidromic response that occurred at a spike-stimulus interval of 1.1 ms (antidr; ***G***); this was collided at an interval of 1 ms (***F***).

The recording arrangement is shown schematically in Figure 1*A*. We performed a craniotomy over the left and right motor cortices, including M1 and SMA. The dura was removed to expose the cortical surface. A custom stimulating electrode consisted of a flexible plastic tube, from which eight silver wires exited. These wires were insulated apart from at their ends, which were formed into a small loop around 3 mm in diameter. The tube was fixed to the skull near to the craniotomy using skull screws and dental acrylic; the wire loops were then placed to be in gentle contact with the cortical surface. Three wires were each positioned over M1 on each side, at ∼6, 10, and 16 mm lateral to the midline to target the leg/trunk, arm/forearm and hand representations. We refer to these locations as “medial,” “middle,” and “lateral” in the text and figures. Two further wire loops were positioned over the left and right SMA. The whole assembly was then covered in Vaseline and gauze to prevent tissue drying while ensuring that electrodes were not short-circuited to each other.

A laminectomy was performed exposing cervical spinal segment C4. A small craniotomy of the occipital bone was also created, extending 5 mm bilaterally, dorsal to the foramen magnum. The dura underneath this window was removed and the cisterna magna was opened, exposing obex and the dorsal surface of the brainstem. Where necessary to visualize the brainstem better, the caudal part of the cerebellum was gently reflected by placing a small piece of cotton wool between the cerebellum and the occipital bone bilaterally. A mineral oil pool was constructed to prevent cooling or desiccation of the exposed brainstem and spinal cord.

The anesthetic regime was then switched to an intravenous infusion of midazolam (260–740 µg kg^−1^ h^−1^), ketamine (5–13 mg kg^−1^ h^−1^) and alfentanil (19–71 μg kg^−1^ h^−1^). This was supplemented with a low dose of sevoflurane (0.8%) in one animal, otherwise inhalational anesthetic was discontinued as we have found that this regimen leaves the central nervous system more active, while providing stable deep anesthesia. The vertebral column was clamped at high thoracic and mid-lumbar levels. The head was fixed stereotaxically and angled to produce ∼70° neck flexion. Before single unit recordings, neuromuscular blockade was commenced (atracuronium, initial dose of 0.7 mg kg^−1^ followed by 0.7 mg kg^−1^ h^−1^). A bilateral pneumothorax was made to minimize respiratory movements. Continuous monitoring of a broad range of physiological parameters (including blood pressure, oxygen saturation, heart rate, end-tidal CO_2_, and core temperature) ensured deep anesthesia and a good physiological condition. Slowly rising trends in heart rate or blood pressure, or more rapid increases in response to a noxious stimulus, were taken as evidence of waning anesthesia; supplemental doses of the injectable agents were then given, and infusion rates increased accordingly.

Two pairs of parylene-insulated stainless steel-stimulating electrodes (MS501G, Microprobe Inc) were inserted in the C4 spinal segment to allow antidromic identification of reticulospinal cells, one pair on each side. Electrodes were inserted 1.5 mm lateral to the midline, 4.7 mm below the cord surface, and 2.3 mm lateral to the midline, 4.3 mm below the cord surface. Bone screws were inserted into the lateral mass of the C4 vertebra on each side, and a steel rod fixed between them. Each electrode was inserted using a micromanipulator; once in position, it was fixed to this rod using dental acrylic, and the manipulator removed. Stimulation (up to 1 mA, biphasic pulses, 0.2 ms per phase) through each electrode was referenced to a needle inserted in the nearby paraspinal muscles.

Use of surface cortical stimulation has the advantage of generating robust activation, but it is important to consider the extent to which the stimulus spreads. Before the commencement of neuromuscular block, we stimulated through each electrode with a train of 18 pulses and noted the motor threshold and movement elicited. Thresholds varied from 1.4 to 9.2 mA; this is comparable to motor thresholds measured in human patients with epicortical grid electrodes over M1 (2–7 mA; Hiremath et al., 2017). Movements were in accord with the known cortical representation, with leg or trunk, arm or forearm and hand movements seen from the medial, middle and lateral M1 electrodes, respectively. This argues against extensive spread, as, for example, no leg effects were seen from the middle or lateral electrodes. Stimulation of the SMA produced bilateral finger movements; no leg movements were seen from SMA stimulation, again arguing against spread to the adjacent M1 leg representation. In one animal, we also recorded surface volleys from the C6 spinal segment after single-pulse stimulation of each cortical electrode. At the intensity of 5 mA used in almost all recordings, a clear direct (D) volley was produced from all sites, with a smaller indirect (I1) wave from some electrodes. The latency to the first negative inflection of the D wave was 1.3 ms. This is compatible with activation at the cortical surface; spread to the white matter would produce a volley around 0.5 ms earlier, based on previous work (Edgley et al., 1990). A final argument against extensive stimulus spread was that we commonly found quite different responses in our single cell recordings in response to stimulation of adjacent cortical electrodes.

At the end of the experiments, animals were killed with an overdose of anesthetic.

#### Intracellular recordings

Three animals were used for this part of the experiment. Intracellular recordings were made from neurons in the reticular formation using sharp glass micropipettes (tips broken to give impedance of 5–25 MΩ) filled with 2 m potassium acetate and connected to a bridge amplifier (BA-03X, NPI). The arachnoid was removed, and electrodes inserted into small patches teased in the pia with watchmaker's forceps. Recording stability was improved using a pressure foot which gently pressed on the brainstem near to the penetration. The electrode was advanced rapidly using a piezoelectric drive (Burleigh PCS-6000, Thorlabs) while observing the responses following stimulation of one of the spinal electrodes. An extracellular antidromic field indicated that the tip was close to reticulospinal cells; movements were then made in 2-µm steps, to allow location and penetration of a reticulospinal cell antidromically activated from the spinal cord.

In some cases, cells fired spontaneously, and this made measurement of synaptic responses difficult; we applied hyperpolarizing current through the bridge amplifier to prevent this firing. Typically, the level of current could be gradually reduced during the recording as the electrode seal improved.

Once a stable intracellular recording had been obtained, we recorded the synaptic responses to single stimuli and trains of up to four stimuli (3-ms interstimulus interval) applied to the cortical electrodes (biphasic pulses, 0.2 ms per phase, negative phase first, 4-Hz repetition rate, intensity typically 5 mA, although 10 mA used for some cells in monkey S). Isolated constant-current stimulators (Model DS4, Digitimer or Model 2100, A-M Systems Inc) were used to deliver all stimuli; these stimulators were connected to the animal via computer-controlled relays which allowed each electrode to be activated in sequence automatically. A silver ball electrode on the brainstem surface close to the electrode penetration point recorded surface volleys simultaneously with the intracellular potentials (Fig. 1*B*). Intracellular (gain 10, DC-10-kHz bandpass) and epidural recordings (gain 10,000, 30 Hz to 10 kHz or 300 Hz to 7.5 kHz bandpass) were sampled to hard disk via a Power1401 interface and Spike2 software (Cambridge Electronic Design Ltd, 25,000 samples/s) for off-line analysis.

#### Extracellular recordings

Intracellular measurements have the great advantage of revealing sub-threshold activation by weak connections; however, successfully penetrating reticulospinal cells and maintaining intracellular conditions meant that the recording yield was low. We therefore supplemented our dataset with a larger number of extracellular recordings in two further animals (monkeys A and Sh). These recordings used an Eckhorn microdrive (Eckhorn and Thomas, 1993), with which we have successfully recorded from the reticular formation in anaesthetized animals previously (Fisher et al., 2012). The microdrive was loaded with four glass-insulated tetrodes; penetrations were made at a 45° angle into the reticular formation through the craniotomy adjacent to the foramen magnum similar to the intracellular recordings. Obex was used as the primary landmark; penetrations were made 0–2.5 mm rostral and 1–2 mm lateral to obex. As for the intracellular recordings, spinal stimulation was used as a search stimulus to detect when the advancing electrodes first encountered reticulospinal cells. We then searched for clean single units that were antidromically activated by stimulation through the spinal electrodes. The activation was determined to be antidromic based on a very low (<0.15 ms) jitter in latency, whereas synaptic activation produced much larger jitter (Fig. 1*D*,*E*). Antidromic effects also had a sharp threshold for all-or-none unit activation. Lack of spontaneous firing often made it difficult to run a collision test, as we routinely do to confirm identification of corticospinal cells by antidromic activation from the pyramidal tract (Baker et al., 1999). In some cases, we were able to make the reticular neuron fire by stimulating one of the cortical electrodes. In this case, the orthodromic spike so produced was used to trigger cord stimulation, allowing performance of a collision test (Fig. 1*F*,*G*). Where this was not possible, antidromic identification was forced to rely on low jitter and sharp threshold alone. Once one or more antidromically-identified units was identified, we recorded the responses to cortical stimulation as for the intracellular recordings. Recordings were typically made for 400 s; with a stimulus rate of 4 Hz, and eight cortical electrodes activated with between one and four stimuli, this usually gave 50 sweeps per condition, although in some cases recordings were lost earlier than this.

#### Analysis

Postsynaptic responses to stimuli were identified from the intracellular recordings. Synaptic delays were measured from the first inflection in the epidural volley to the onset of the EPSP or IPSP. The amplitude of postsynaptic potentials was measured from onset to peak. Resting membrane potential was estimated as the most negative voltage encountered after penetrating the cell. This was corrected by the offset voltage measured when the electrode left the cell and moved into the extracellular space at the end of the recording. Measurements of membrane potential were only made in cells with stable recordings, in situations where no hyperpolarizing current was needed to prevent continuous cell firing.

Antidromic responses were displayed on a computer screen, and the antidromic latency measured to the first deflection from baseline. This raw latency was corrected by the utilization time by subtracting 0.1 ms, being half the width of the negative phase of the spinal stimulus. Conduction distance was measured from the spinal stimulation electrodes to the recording site in the brainstem; conduction velocity was then estimated by dividing the distance by the corrected antidromic latency. In monkey S no conduction distance measures were available. In the other two animals used for intracellular recording measured distances were the same to within 1 mm; as we aimed for the same stimulation and recording sites in monkey S, and the animals were a similar weight, we used the same measurement also for monkey S. Some cells were lost after measuring responses to antidromic stimulation, but before sufficient data on responses to cortical stimulation were gathered; these cells are included in the description of conduction velocities only.

Extracellular recordings exhibited substantial artefacts following the cortical stimulation, which if uncorrected could interfere with the spike discrimination process. The raw recording file was therefore first processed by digitally subtracting an estimate of the artifact generated by averaging, as described in Koželj and Baker (2014). Times of single unit firing were then discriminated using custom-written clustering software (GetSpike; S.N.B.). Subsequent analysis involved compiling peristimulus time histograms (PSTHs) relative to the different stimulus markers, with a bin width of 0.1 ms. The experimenter marked the onset and offset of putative responses using interactive cursors, and the significance of this response was assessed by computing:

$$Z=\frac{\frac{C_{p}}{N_{p}}-\frac{C_{b}}{N_{b}}}{\sqrt{\frac{C_{p}}{N_{p}^{2}}+\frac{C_{b}}{N_{b}^{2}}}},$$

where *C_p_* and *C_b_* are the number of counts in the response peak and baseline regions, respectively, and *N_p_* and *N_b_* are the width of the peak and baseline regions in bins. The baseline region was taken as the 5 ms preceding the first stimulus. Under the null hypothesis that the firing rate in the baseline and response regions is the same, Z will be approximately normally distributed with mean zero and standard deviation one (Cope et al., 1987). Peaks with |Z| > 3.29 were considered significant, corresponding to *p* < 0.001 (two tailed). This conservative significance level was chosen to correct for the implicit multiple comparisons involved in selecting the best region to test; assignment of peak significance accorded well with judgements made by eye. The amplitude of responses *s* was measured as the number of excess spikes above baseline elicited per stimulus:

s=Cp-NpCbNbNs,

where *N_s_* is the number of stimuli (Abeles, 1982). The precarious nature of recordings (especially intracellular) meant that cells were sometimes lost before all measurements could be made. In Results, data are reported from all cells from which a given measurement was available, together with the relevant number of cells.

#### Reconstruction of recording sites

Brainstem tissue from monkeys A and Sh was retained for histologic verification of recording sites. Tissue was sectioned on a freezing microtome at 40 µm, and free-floating sections were stained with cresyl violet. Tiled high-magnification images were acquired, and traced in a drawing package (Coreldraw); penetration maps based on electrode coordinates were then overlain.

## Results

### Intracellular recordings

**Figure 2.**
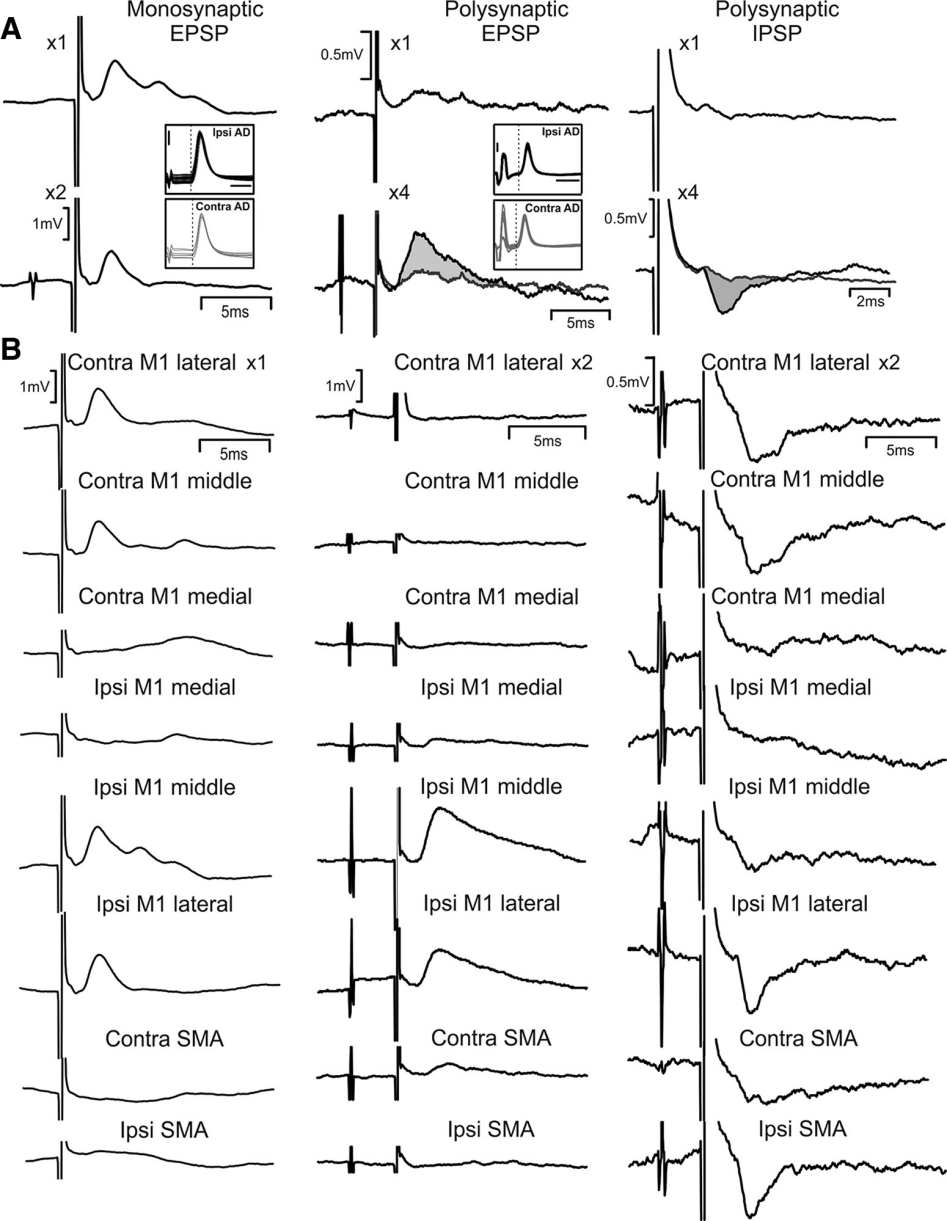
Example intracellular recordings. ***A***, Examples in three different cells to show the different types of synaptic response. Left column, Synaptic delay 0.97 ms, and potential does not grow with a pair of stimuli (×2) compared with a single stimulus (×1), indicating a monosynaptic EPSP. Response to ipsilateral M1 middle stimulation. Middle column, Small response to single stimulus (×1), which grows considerably with four (×4); synaptic delay >1 ms, indicating a oligosynaptic EPSP. Response to contralateral SMA stimulation. Right column, No response to single stimulus; inhibitory response grows with four stimuli (×4), indicating a oligosynaptic IPSP. Response to ipsilateral M1 lateral stimulation. For oligosynaptic responses, sweeps following a single stimulus have been superimposed on responses to four stimuli in thin lines; gray shading highlights the synaptic potential which appears with multiple stimuli. Insets show antidromic responses to spinal stimulation on the side ipsilateral or contralateral to the recording site. Cell in right column could not be antidromically activated. Calibration bars for insets showing antidromic responses are 2 mV, 1 ms. ***B***, Responses to stimulation of each cortical electrode in turn, as indicated, for the same cells illustrated above in ***A***.

Recordings were made from 64 antidromically identified reticulospinal neurons in the three monkeys used for this part of the study. The mean resting membrane potential was estimated as −43.9 mV (SD 14.1 mV, range −83.2 to −27.2 mV; *n* = 22 recordings where membrane potential measurement was possible; see Materials and Methods). Figure 2 shows example intracellular records following cortical stimulation. The response in the left column of Figure 2*A* (from stimulation of ipsilateral M1) had a short latency, only 0.97 ms longer than the surface volley recorded near to the intracellular electrode. It did not grow following the second stimulus of a train (but was actually 17% smaller). Accordingly, this is likely to be mediated by a direct monosynaptic connection. By contrast, the response in the middle column of Figure 2*A* following a single stimulus to the contralateral SMA (top trace) was very small (160 μV amplitude), and had a delay from the volley of 1.02 ms. The EPSP grew with successive stimuli in a train, until it was 610 μV in amplitude after the fourth (bottom trace), a rise of 280%. These are properties indicative of a disynaptic or oligosynaptic response.

Most synaptic responses observed in reticular cells following cortical stimulation were excitatory, although there was sometimes evidence that an initial excitation was followed by inhibition, for example, the EPSP following two stimuli to the ipsilateral M1 lateral electrode shown in the left column of Figure 2*B* had a very rapid falling phase, consistent with a later IPSP. Occasionally an overt initial IPSP could be seen, as illustrated in the right column of Figure 2*A*, usually as in this case when not superimposed on an EPSP. Little response was observed following a single stimulus to the ipsilateral M1 (top trace), but an IPSP appeared in response to a train of four (bottom trace). As corticofugal fibers are glutamatergic (excitatory), any inhibitory effects would have to be mediated via at least one interposed interneuron. This is consistent with the observed growth of the IPSP with a stimulus train.

Figure 2*B* illustrates responses from all cortical stimulating sites tested, in the cells presented in Figure 2*A*. There is clearly considerable convergence: for example, the cell with monosynaptic EPSPs (Fig. 2*B*, left column) received input from two ipsilateral M1 sites, two contralateral M1 sites, but not from SMA.

**Figure 3.**
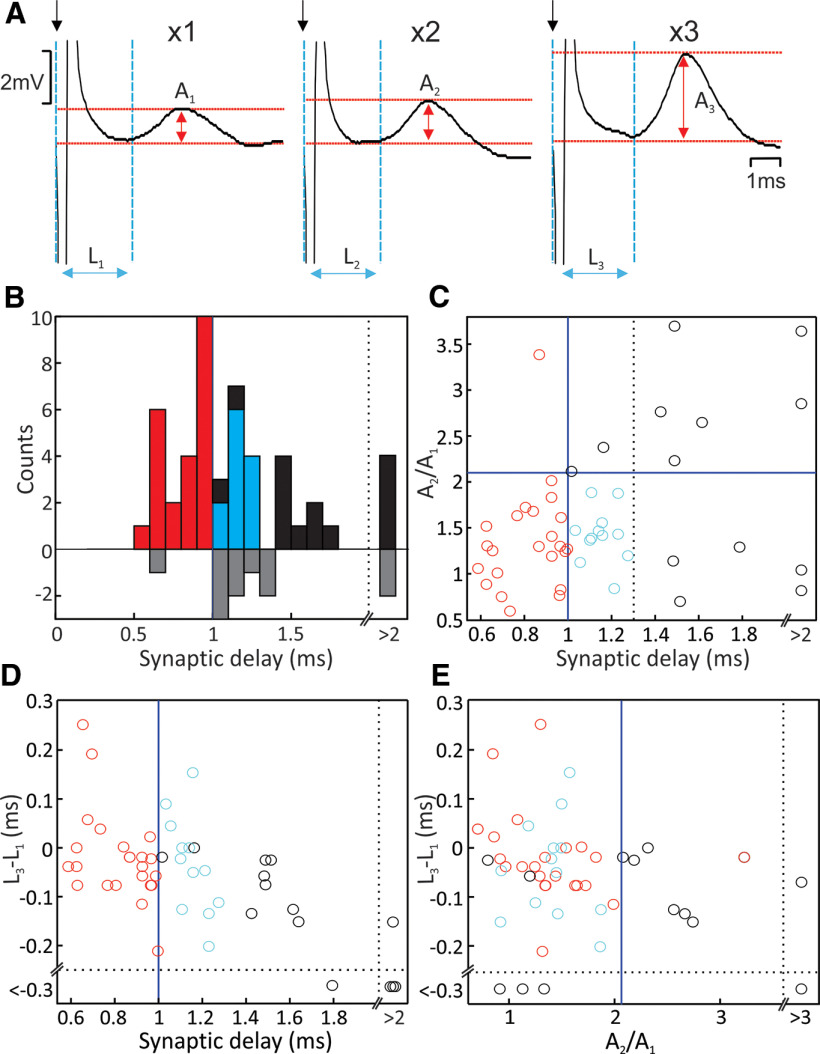
Classification of responses as monosynaptic. ***A***, Example averaged EPSPs to increasing numbers of stimuli applied to the same cortical electrode. Amplitudes and latencies for responses to multiple stimuli were estimated by subtracting the trace with one fewer stimuli, leaving the extra response evoked by the last stimulus in the train. ***B***, Histograms of EPSP synaptic delay. Upward bars show measurements from responses to a single cortical stimulus; downward bars from responses which only became apparent with multiple stimuli. Remaining plots relate only to the responses visible to one stimulus. ***C***, Scatter plot of augmentation ratio (A_2_/A_1_; see panel ***A***) versus synaptic delay. ***D***, Latency shortening (L_3_-L_1_; see panel ***A***) versus synaptic delay. ***E***, Latency shortening versus augmentation ratio. Blue lines in ***B–D*** indicate thresholds used to exclude responses as non-monosynaptic. Points excluded on the basis of too large an augmentation ratio, or synaptic delay >1.3 ms, are colored black. Points with synaptic delays shorter than 1 ms and hence very likely to be monosynaptic are colored red. Cyan points have synaptic delays longer than 1 ms, but augmentation ratios and latency shortening comparable to the red points; these are accepted as likely to be monosynaptic, but mediated via slower corticoreticular fibers.

Figure 2 makes it clear that responses compatible with both monosynaptic or oligosynaptic mediation were seen. Determining the synaptic linkage underlying these responses is not straightforward. The usual approach is to use a short and fixed latency and a lack of facilitation with a train of two stimuli to argue for a monosynaptic pathway. These approaches are complicated here because the cortical stimulation could activate cortical output neurons directly or indirectly (Patton and Amassian, 1954), and the cortical output neurons have a wide potential range of different conduction velocities. It is also known that reticulospinal neurons can be activated by local circuits within the reticular formation itself (Edgley et al., 2004). Figure 3 presents the approach which we took to classifying responses in an objective way, similar to our previous work (Witham et al., 2016). The amplitude and onset latency of the EPSPs were measured following one, two and three stimuli (Fig. 3*A*). Figure 3*B* shows the distribution of synaptic delays. Bars plotted upwards relate to responses visible following only a single stimulus; those plotted down relate to the smaller number of EPSPs which only appeared clearly after stimulus trains (Fig. 2*A*, middle column). More than one synapse is likely to mediate these responses, although it remains possible that these are produced from a monosynaptic response in the reticular formation following an indirectly-elicited corticofugal volley. For the responses elicited by a single stimulus, more consideration is required, and two further measures were made.

First, we measured the augmentation ratio, as the amplitude of the EPSP following the second of two stimuli divided by the amplitude following only one stimulus (A_2_/A_1_; Fig. 3*A*). Second, we measured the reduction in latency of the EPSP following the third stimulus of a train, compared with the first (L_3_-L_1_; Fig. 3*A*). Monosynaptic responses should change little in amplitude or latency with a stimulus train, whereas oligosynaptic EPSPs should grow in amplitude and shorten in latency with successive stimuli. Figure 3*C–E* show pairwise scatter plots of augmentation ratio, latency shortening and synaptic delay.

Responses with synaptic delay <1 ms are very unlikely to be mediated by more than one synapse (Jankowska et al., 2003); these are shown by red points in Figure 3. All but one such EPSP had an augmentation ratio <2.1 (Fig. 3*C*, horizontal line). It is known that monosynaptic connections can show facilitation in this range (Porter, 1970). We accordingly took this as the upper limit compatible with monosynaptic responses, and rejected longer latency EPSPs with larger augmentation ratios as possibly oligosynaptic (black points above the horizontal blue line). EPSPs with synaptic delay <1 ms had latency changes with successive stimuli which spanned the full range observed in the population (Fig. 3*D*), suggesting that latency change was not useful in discriminating putative monosynaptic versus oligosynaptic responses here. The distribution of augmentation ratio and latency shortening was very similar for the unambiguous monosynaptic responses with synaptic delay <1 ms (red), and for points with synaptic delays 1–1.3 ms (Fig. 3*C*). All but two points with synaptic delays 1–1.3 ms has augmentation ratios <2.1 (Fig. 3*C*). We therefore consider that these were most likely also to be mediated monosynaptically, via corticoreticular fibers with slower conduction velocity. In the following analysis, EPSPs with synaptic delays between 1 and 1.3 ms and augmentation ratios <2.1 have been classified as of monosynaptic origin (Fig. 3, cyan points), as well as those with delays <1 ms. We chose this cutoff to be conservative; it is possible that the few points with longer delays and low augmentation ratios may also have been monosynaptically mediated. It is also possible that responses with high augmentation ratios contained mixed monosynaptic and oligosynaptic components.

**Figure 4.**
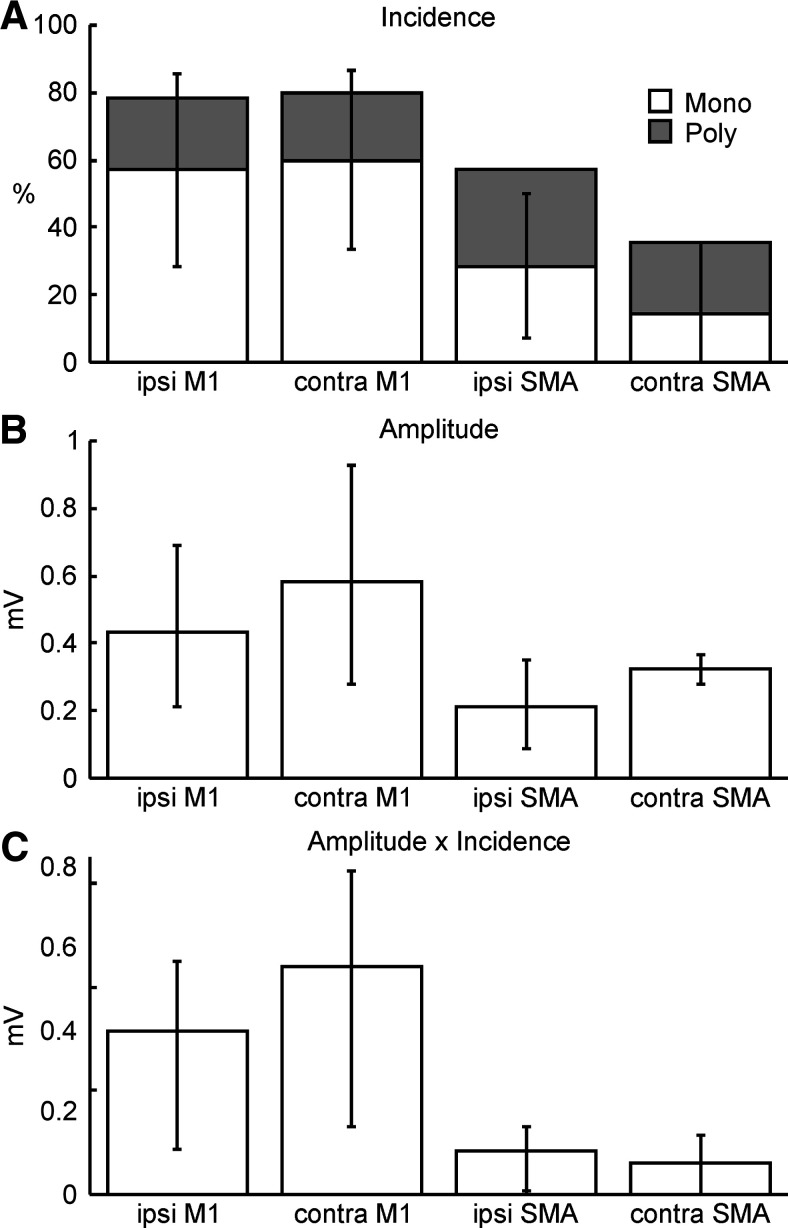
Properties of responses in intracellular recordings from antidromically-identified reticulospinal neurons. ***A***, Incidence of EPSPs following stimulation of different cortical sites, classified as monosynaptic (white) or oligosynaptic (gray). ***B***, Amplitude of EPSPs, measured only from cells with monosynaptic responses. ***C***, Amplitude × incidence, also only for monosynaptic responses, providing an overall measurement of the efficacy of direct input from a given cortical area. Error bars show 95% confidence intervals on the measure, estimated used a Monte Carlo resampling technique. Error bars in ***A*** relate to the monosynaptic incidence.

Figure 4 shows measurements made from the synaptic responses to cortical stimulation in antidromically-identified RST cells (based on *n* = 11 cells, except for responses to contralateral M1 where *n* = 12). All cells could be activated from at least one cortical area. Figure 4*A* presents the incidence of EPSPs from each source, separated by the classification of effects as monosynaptic versus oligosynaptic as discussed above. Figure 4*B* shows the average amplitude of the monosynaptic EPSPs; for M1, where three cortical sites were stimulated, the largest EPSP in a given cell has been used to compile this estimate. Figure 4*C* shows the amplitude × incidence, equivalent to the product of the values given in Figure 4*A*,*B*. This provides an overall estimate of the size of the effect of cortical stimulation; it is equivalent to measuring average amplitude, including (as zeros) sites without EPSPs (Zaaimi et al., 2012). There was no significant difference in EPSP amplitude for the different stimulation sites (one way ANOVA, *F*_(3,19)_ = 0.78; *p* = 0.517).

**Figure 5.**
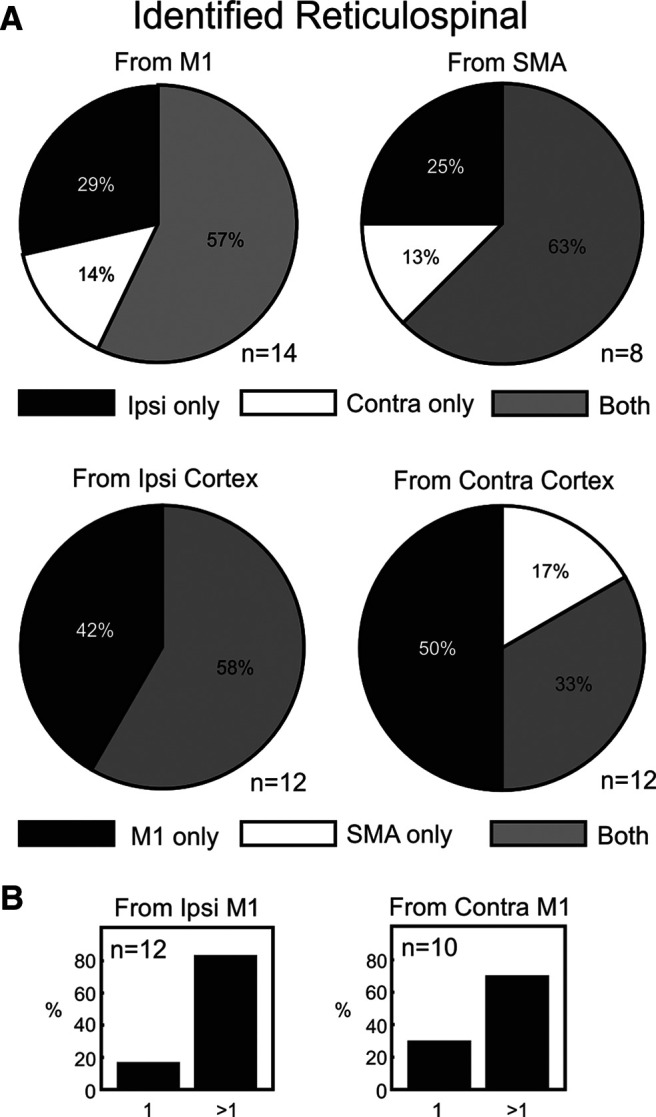
Fraction of responses observed in intracellular recordings of reticulospinal cells from different cortical stimuli. ***A***, Proportions of reticulospinal neurons receiving either monosynaptic or oligosynaptic input from only one site of a pair, or both. Top row shows convergence between the two hemispheres from the same cortical area (M1 or SMA). Bottom row shows convergence between different cortical areas within the same hemisphere. For each plot, cells have been excluded if they showed no response to either stimulus. ***B***, Convergence between sites within M1 in the same hemisphere. Each bar chart indicates the proportion of cells which receive input from just one, or more than one, M1 site.

Figure 5 summarizes the origin of inputs to individual reticulospinal cells from our intracellular dataset. Cells which received no relevant inputs were excluded from these plots, so the analysis is based on relatively small numbers (*n* = 8–14 units). Connections have been counted for this plot, regardless of their putative synaptic linkage (monosynaptic vs oligosynaptic). The top row shows convergence from the same area; the bottom row, convergence between M1 and SMA within the same hemisphere. Despite the low number of cells for analysis, there is still evidence of convergence from multiple cortical sites. Figure 5*B* looks more specifically at convergence from multiple sites within M1 in the same hemisphere, by showing the proportion of cells which received input from only one of the three available surface-stimulating electrodes, or more than one. We found that between 70% and 83% of cells received input from more than one cortical site. Given that the spacing between the cortical electrodes was around 5 mm, this indicates that a large part of the M1 representation could project to a given reticular neuron.

### Extracellular recordings

**Figure 6.**
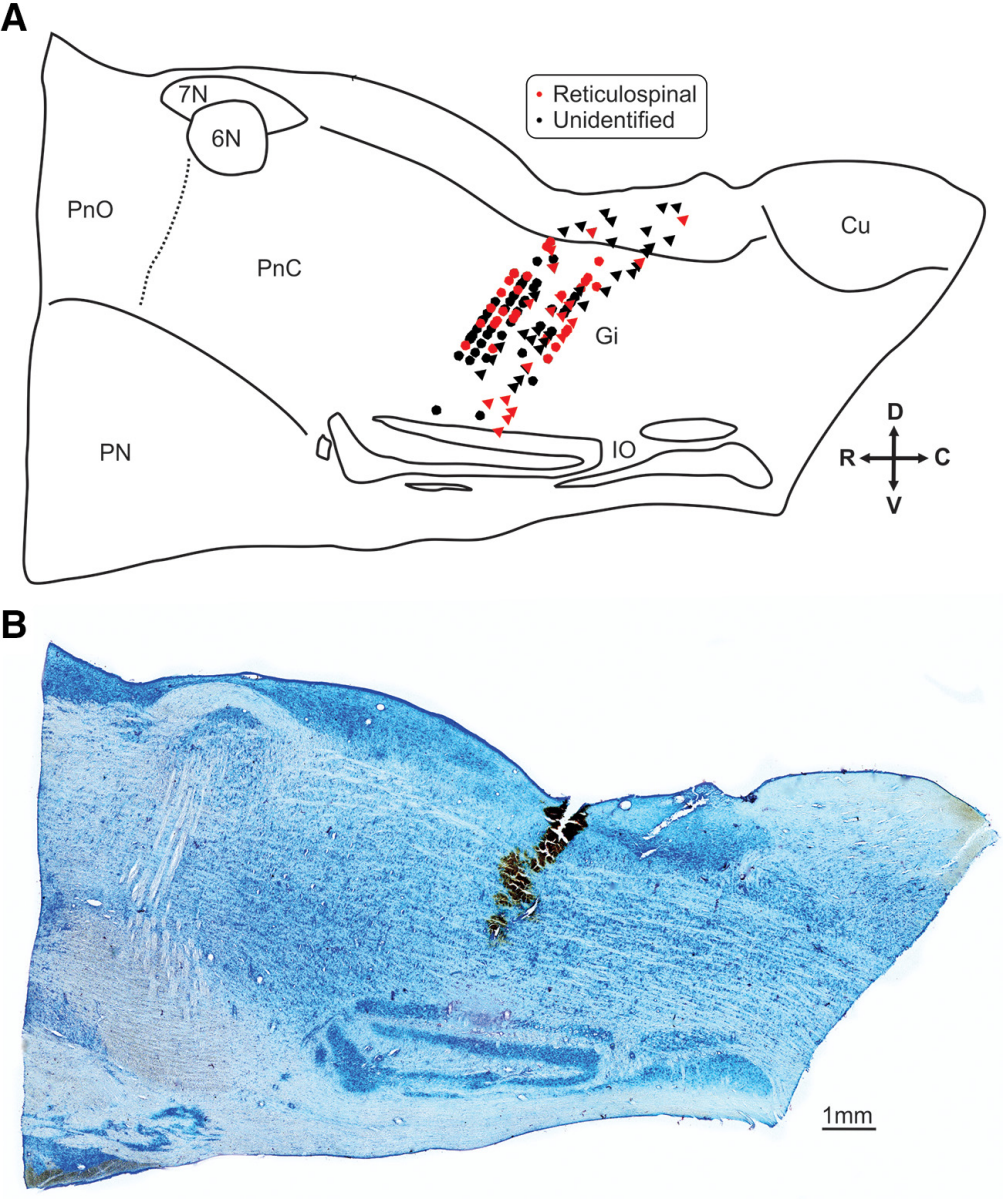
Reconstruction of extracellular recording sites. Recordings were made 1–2 mm left or right of the midline in two monkeys. ***A***, Sites from both sides and the two animals have been combined and plotted together in the parasagittal plane. The reconstructed recording locations are superimposed onto a representation of the macaque brainstem anatomy, traced from a tissue section from monkey A. Circles denote recording sites from monkey A and triangles are from monkey S. Brainstem structures are labeled as the following: PnO, pontine reticular nucleus oralis; PnC, pontine reticular nucleus caudalis; Pn, pontine nuclei; 6N, abducens nucleus; 7n, facial nerve; Gi, reticular nucleus gigantocellularis; IO, inferior olive; Cu, cuneate nucleus. Arrows at bottom right provide orientation: D, dorsal, V, ventral, R, rostral, C, caudal. ***B***, Representative Nissl-stained section from monkey A showing the location of an ink injection made as along an electrode track after the final recording. An identified reticulospinal cell was recorded at the deepest point of this track which lies within the nucleus gigantocellularis.

Recordings were made from 46 antidromically-identified reticulospinal neurons, and 105 unidentified cells in the two monkeys used for this part of the study. Figure 6*A* shows a reconstruction of the recording sites, mapped relative to the obex landmark in a parasagittal plane. Recordings were made 1–2 mm lateral to the midline on both sides; sites have been combined across sides and animals for this reconstruction. The map has been superimposed on a parasagittal tracing from a histologic section of the brainstem in one animal. It is clear that recording sites were located within the nucleus gigantocellularis. All cells were located in the vicinity of identified reticulospinal neurons (Fig. 6*A*, red points), suggesting that the locations plotted just above and below the gigantocellularis are most likely to reflect minor errors in the reconstruction, rather than off-target recordings. Figure 6*B* shows an example parasagittal brainstem section, in which an ink track is visible which was made along one of the electrode penetrations.

**Figure 7.**
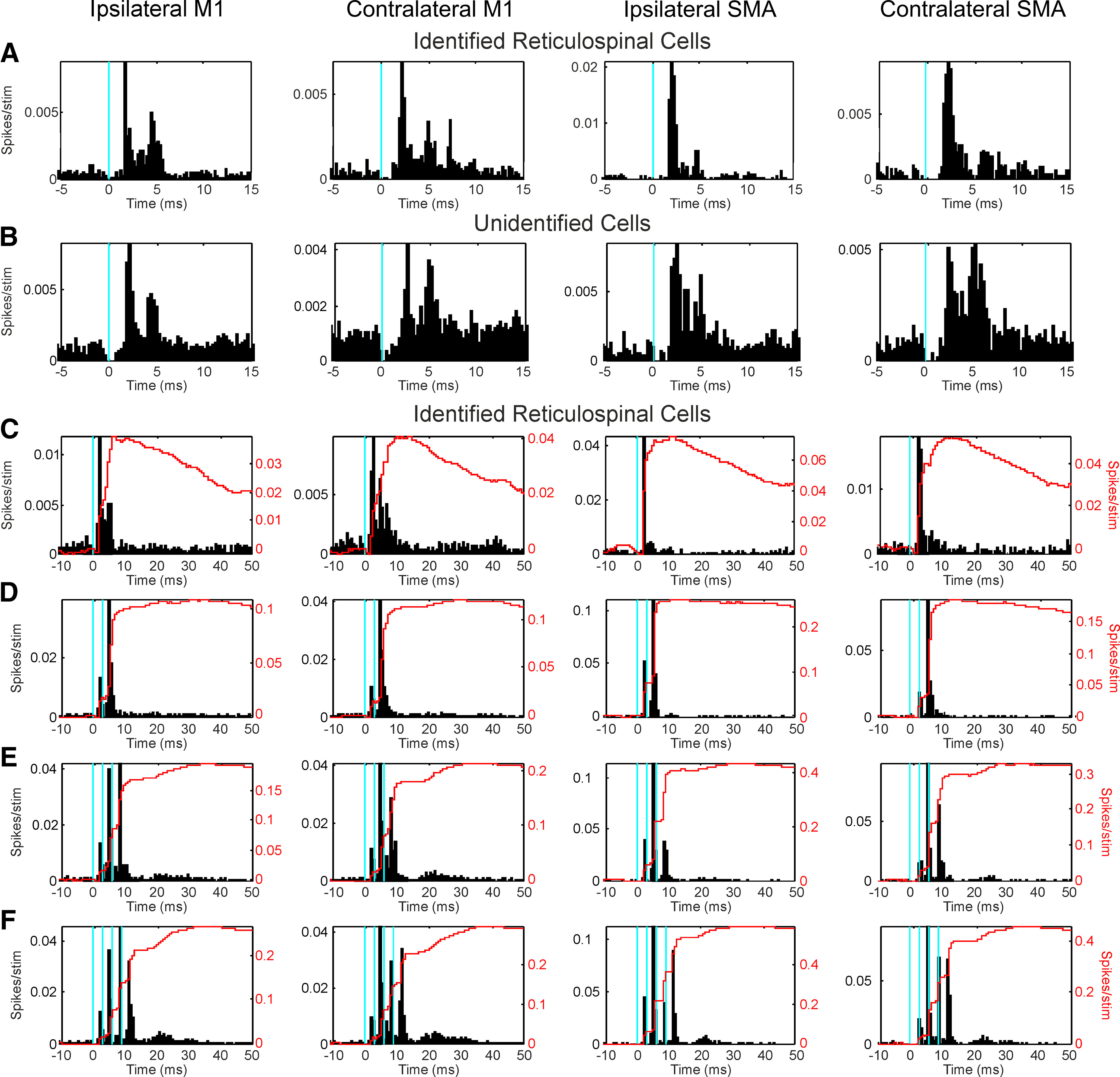
Summed responses to cortical stimulation recorded extracellularly. Each row shows PSTHs, averaged over all available cells within a given class. Each column presents data for a given cortical stimulation site; results for M1 have been averaged over all available M1-stimulating electrodes in one hemisphere. Blue vertical lines mark the times of cortical stimuli. ***A***, Reticulospinal cells. ***B***, Unidentified cells, responding to single cortical stimuli. ***C–F***, Reticulospinal cells responding to between one (***C***) and four (***F***) cortical stimuli, on a longer time scale to emphasize later components of the response. Overlain on the PSTHs are CUSUMs (red lines), plotted against the ordinate on the right of each plot. Bin width: 0.25 ms (***A***, ***B***) and 0.5 ms (***C–F***). Averages compiled from 46 reticulospinal cells and 105 unidentified cells.

Figure 7 presents a summary of the responses to cortical stimulation which were observed. All plots in this figure are averaged PSTHs. Each column shows data from stimulation of a different cortical site as indicated at the top (M1 or SMA, from the hemisphere ipsilateral or contralateral to the recorded neuron). For M1, PSTHs have been averaged across the three available stimulation sites on one side. All PSTHs have been averaged across the available cells in a given category (reticulospinal or unidentified cells); all cells have been included, regardless of whether they were individually assessed as responding to the stimulus or not.

Figure 7*A* presents population data for reticulospinal cells responding to a single cortical stimulus (delivered at the vertical blue line). There was a clear short-latency peak for all four cortical sites with onset around 1.6 ms poststimulus; as for the intracellular data, this indicates that there was a monosynaptic component to the corticoreticular responses. However, in addition to this early peak, there was also a second peak in the averaged PSTH starting around 3.6 ms after the stimulus, which was clearest for the responses to stimulation of M1.

Figure 7*B* presents similar plots for the population of unidentified reticular formation cells. These also showed an early facilitation following stimulation of all four cortical sites, and a second later peak. The unidentified neurons exhibited the second facilitation following all four cortical locations.

Figure 7*C–F* shows results for the reticulospinal neurons for a longer poststimulus time, and for varying numbers of stimuli in a train [single stimulus (Fig. 7*C*) up to a train of four stimuli (Fig. 7*F*)]. Unsurprisingly, each stimulus within a train was followed by the short latency facilitations illustrated in more detail in Figure 7*A*. However, in addition these plots indicate that there was a third component to the responses, which was broader and of even longer latency (peak around 20 ms; Fig. 7*E*,*F*). This component was not visible following a single stimulus, but grew and lengthened following stimulus trains. Overlain on the PSTHs of Figure 7*C–F*, red lines, are CUSUM plots (Ellaway, 1978), which provide an accumulated count of the average number of extra spikes above baseline elicited by the stimulus. The CUSUMs are useful in revealing visually the relative sizes of early versus late responses. The late responses were most important after a train of four stimuli, when they contributed 19.5%, 22.6%, 9.4%, and 12.6% of the total response after stimulation of ipsilateral M1, contralateral M1, ipsilateral SMA, and contralateral SMA, respectively. Although late peak responses are shown only for reticulospinal cells in Figure 7*C–F*, similar results were seen for the unidentified cells (data not shown).

**Figure 8.**
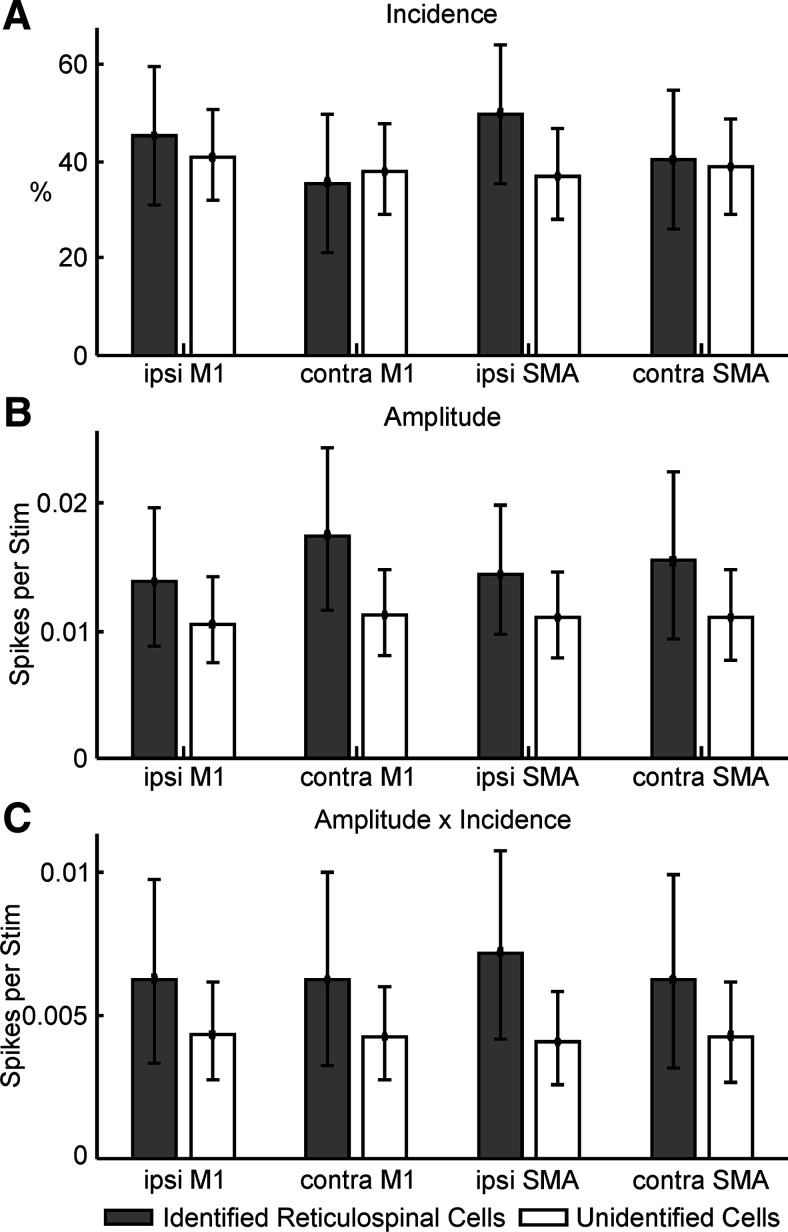
Properties of responses in extracellular recordings. ***A***, Incidence of significant responses following stimulation of different cortical sites. ***B***, Amplitude of responses (calculated as extra spikes produced above baseline per stimulus), measured only over cells with significant responses. ***C***, Amplitude × incidence, providing an overall measurement of the efficacy of a given input. Results in all plots are shown separately for reticulospinal and unidentified cells. Error bars show 95% confidence intervals on the measure, estimated used a Monte Carlo resampling technique.

Figure 7 shows PSTHs averaged across cells, which provides a useful overall summary of responses at the population level. Figure 8 presents results from measurement of responses in single cells. To compile this plot, the largest response has been used from stimulation of a given cortical area, taken across numbers of stimuli in the train, and (for M1) the different electrodes placed over the cortical surface. Only cells tested with a 5-mA cortical stimulus intensity have been included, to ensure an unbiased comparison of responses. Figure 8*A* shows the incidence of statistically significant responses. Overall, across all categories shown, 80/143 (56%) of cells showed significant changes in firing following stimulation of at least one cortical site. Figure 8*B* presents the average amplitude of responses (measured as excess spikes per stimulus *s*; see Materials and Methods) in the same format. Cells have only been included here if responses were significantly different from zero. Two way ANOVA indicated a significant effect on amplitude of cell type (reticulospinal greater than unidentified cell; *F*_(1,219)_ = 6.93, *p* = 0.0091), but not cortical stimulation site (*F*_(3,219)_ = 0.3, *p* = 0.827) or their interaction (*F*_(3,219)_ = 0.16, *p* = 0.925). The average amplitude across all categories was *s* = 0.0123, suggesting that one extra spike was generated on average for every 81 stimuli. Given the reduced excitability of our anesthetized preparation, this likely reflects a strong synaptic connection.

Figure 8*C* presents a plot of incidence × amplitude (as shown for intracellular data in Fig. 4*C*). Repeated measures ANOVA on EPSP amplitude (counting cases where there was no response as zero amplitude) showed no significant effect of cell type (*F*_(1,141)_ = 1.82, *p* = 0.180), cortical stimulation site (*F*_(3,423)_ = 0.169, *p* = 0.917) or their interaction (*F*_(3,423)_ = 0.459, *p* = 0.711).

**Figure 9.**
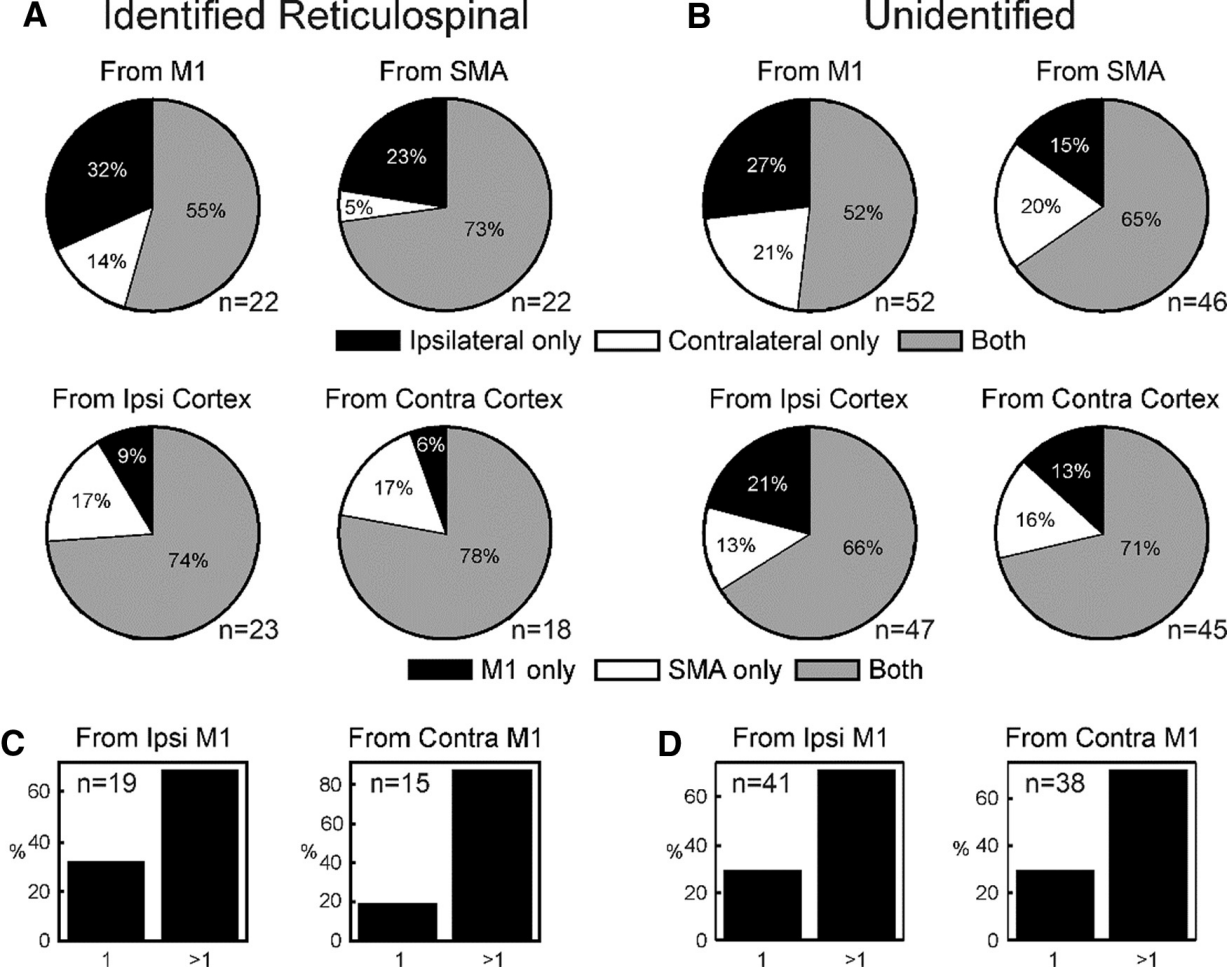
Convergence of different inputs to single reticular formation neurons. ***A***, Proportions of reticulospinal neurons receiving monosynaptic or oligosynaptic input from only one site of a pair, or both. Top row shows convergence between the two hemispheres from the same cortical area (M1 or SMA). Bottom row shows convergence between different cortical areas within the same hemisphere. For each plot, cells have been excluded if they showed no response to either stimulus. ***B***, As ***A*** but for unidentified cells. ***C***, ***D***, Convergence between sites within M1 in the same hemisphere. Each bar chart indicates the proportion of cells which receive input from just one, or more than one, M1 site. ***C***, For reticulospinal cells. ***D***, For unidentified cells. Number of cells contributing is shown as *n* value for each plot individually.

Figure 9 presents data on how inputs from different cortical sites converged onto single neurons. The analysis has been performed pairwise; for example, the top left pie chart shows the number of cells receiving input from the ipsilateral or contralateral M1 alone, or convergent input from M1 on both sides. Cells which receive no input from M1 are not included in this plot. The top row focuses on convergence from the same area in each hemisphere; the bottom row on convergence between M1 and SMA within the same hemisphere. Results are presented for reticulospinal (Fig. 9*A*) and unidentified cells (Fig. 9*B*) separately. No matter the combination, the results provide evidence of large-scale convergence: at least half of the cells with input from one site of a pair received input from both sites.

Figure 9*C*,*D* extends this analysis to investigate convergence from multiple sites within M1 in the same hemisphere, by showing the proportion of cells which received input from only one of the three available surface-stimulating electrodes, or more than one. Depending on the combination of laterality and cell classification, between 68% and 87% of cells received input from more than one electrode. Given that the spacing between the cortical electrodes was around 5 mm, this indicates that a large part of the M1 representation could project to a given reticular neuron.

For identified RST cells, responses were significantly more common from stimulation of the lateral than medial M1 electrode (ipsilateral M1: 42% vs 23%, *p* = 0.013; contralateral M1: 35% vs 21%, *p* = 0.041, McNemar test for proportions). By contrast, lateral and medial M1 stimulation produced responses in unidentified cells with similar frequency (ipsilateral M1: 35% vs 27%, *p* = 0.099; contralateral M1: 26% vs 27%, *p* = 1.000).

For 36 identified reticulospinal cells, we were additionally able to check whether they could be antidromically activated from the spinal electrodes implanted ipsilateral or contralateral to the cell body. We found that nine, four, and 23 cells could be activated from the ipsilateral cord only, contralateral cord only, or from both sides, respectively (equivalent to 25%, 11%, and 64%). There were 14 cells with M1 input which could be antidromically activated from both sides of the cord; three, three, and eight of these cells received input from the ipsilateral side only, contralateral side only and both sides of M1, respectively (equivalent to 21%, 21%, and 57%). There were five cells with M1 input which could be antidromically activated from only one side of the cord; three of these received input from only the ipsilateral hemisphere and two from both sides. Interestingly, this pattern was seen both for cells with exclusively ipsilateral (three cells, two with input from only ipsilateral M1) or contralateral (two cells, one with input from only ipsilateral M1) spinal projections. Similar results were seen for inputs from SMA.

To provide the best overall estimates of convergence, we combined together the intracellular and extracellular datasets. For RST cells which received input from M1, 20/36 (56%) had responses to M1 stimulation in both hemispheres, and 30/36 (=83%) responded to more than one M1 site (regardless of hemisphere). For RST cells which received input from SMA, 21/30 (70%) had responses to SMA in both hemispheres. Of RST cells with any cortical input, 29/37 (78%) had responses to both SMA and M1 (regardless of hemisphere).

### Conduction velocity of reticulospinal neurons

**Figure 10.**
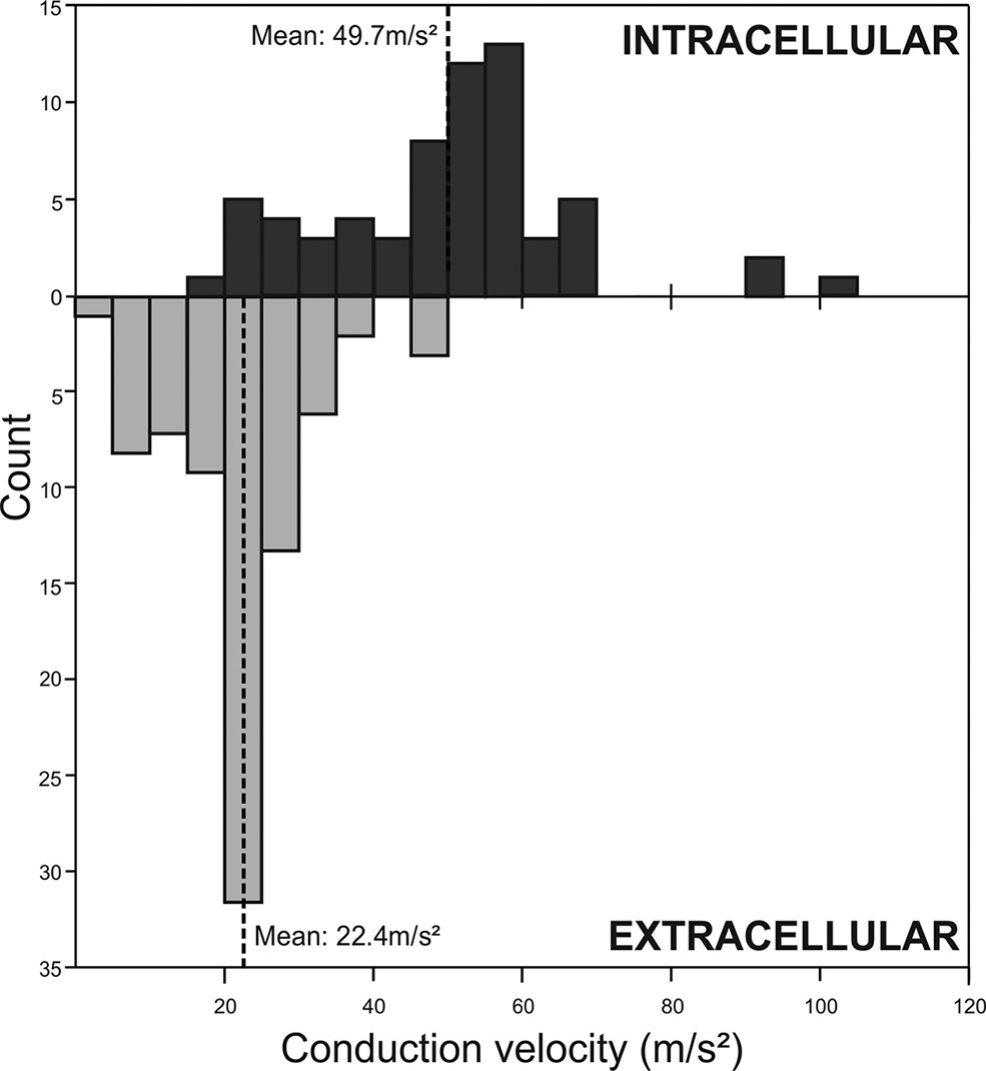
Distribution of reticulospinal conduction velocities. Observed velocities are plotted as histograms for intracellular and extracellular recordings separately. Dotted lines indicate the means of each distribution.

Figure 10 shows histograms of the conduction velocity of the identified reticulospinal cells, determined from the measures of antidromic latency and estimated conduction distance between the spinal stimulation and brainstem recording sites. Results are presented separately for neurons recorded intracellularly and extracellularly (*n* = 64 and 80, respectively). The distribution for the extracellular recordings appeared unimodal, with a mean of 22.4 m/s. By contrast, for the intracellular recordings the distribution appeared bimodal. The lower peak was broadly consistent with that from the extracellular recordings, but in addition there was a second population of cells with faster conducting axons. The overall mean conduction velocity for the intracellular recordings was 49.7 m/s. The faster velocities may relate to the severe bias toward recording from large cells when making intracellular penetrations using sharp microelectrodes.

## Discussion

### Extensive corticoreticular convergence

Our major novel finding is extensive convergence from broad areas of M1 and SMA bilaterally onto neurons in the primate reticular formation, including reticulospinal neurons. Our findings in primates agree with previous work in other species, where extensive convergence from areas within one hemisphere and between hemispheres has previously been demonstrated (Rossi and Brodal, 1956; Magni and Willis, 1964; He and Wu, 1985; Newman et al., 1989; Matsuyama and Drew, 1997; Rho et al., 1997). Recent tract tracing studies in monkey have also revealed bilateral corticoreticular projections from M1, SMA, and lateral PM (Fregosi et al., 2017; Darling et al., 2018), although different combinations of cortical origin and brainstem receiving nuclei may have ipsilateral or contralateral biases in connectivity: our findings show convergence onto individual reticulospinal neurons. This extensive convergence may provide more options for control after cortical damage, such as after stroke. Then, inputs from SMA (Darling et al., 2018) and the contralateral hemisphere (McPherson et al., 2018) may strengthen, restoring some control but also possibly limiting flexibility and hence recovery (McPherson et al., 2018).

The corticospinal projection is strongly lateralized (McNeal et al., 2010; Yoshino-Saito et al., 2010; Soteropoulos et al., 2011; Morecraft et al., 2013), whereas the RST has extensive bilateral projections (Peterson et al., 1975; Davidson and Buford, 2006; Davidson et al., 2007). Sites within the primate reticular formation project predominantly to ipsilateral flexor motor nuclei, and contralateral extensors (Davidson and Buford, 2006). A bilateral corticoreticular projection, in which reticulospinal cells projecting to ipsilateral flexors and contralateral extensors are controlled by the contralateral and ipsilateral hemispheres, respectively, could theoretically give the cortex access to all contralateral motor nuclei. However, such a neat parcellation was not seen. Unlike previous anatomic studies, our electrophysiological measurements were able to reveal extensive convergence to single reticulospinal neurons. Combining across both our intracellular and extracellular datasets, 56% of identified RST cells with input from M1 received it from both sides; for SMA, the equivalent figure was even higher at 70%.

### Direct and indirect corticoreticular connections

In intracellular recordings, many EPSPs appeared compatible with monosynaptic corticoreticular connections (Fig. 3), with mean height 0.45 mV. In extracellular recordings even under anesthesia stimuli could elicit early and frequent responses. The cortex therefore exerts a strong and direct influence over reticulospinal projections.

The second response peak starting 3.6 ms after the stimulus seen in averaged PSTHs compiled from extracellular recordings was 2.0 ms later than the first (presumed monosynaptic) response. This is likely to be mediated disynaptically or polysynaptically, with two possible pathways. Surface electrical stimulation of the cortex elicits an initial direct (D) corticospinal volley, followed by later indirect (I) waves via trans-synaptic activation (Patton and Amassian, 1954). D and I waves are also likely in corticoreticular axons, some of which are corticospinal collaterals (Keizer and Kuypers, 1989): the later peak in the PSTHs may therefore reflect monosynaptic responses within the reticular formation to the first cortical I wave.

Second, the later peak may be generated by intrinsic circuitry within the reticular formation itself. It is known that stimulation of the medial longitudinal fasciculus (MLF) can generate a direct and indirect volley, the latter resulting from excitation of reticulospinal cells by recurrent collaterals (Jankowska et al., 2003; Edgley et al., 2004). The later PSTH peak observed here could therefore reflect recurrent excitation produced by the first peak. A broader third peak was also seen, with onset latency around 8.4 ms after the stimulus. We cannot be certain, but at least part of this could also originate from local reticular circuitry.

We found limited evidence for inhibitory effects. IPSPs were rare in intracellular recordings; summed PSTHs of extracellular discharge showed a net increase in spike count after cortical stimulation, as revealed by CUSUM analysis (Fig. 7). The lack of inhibition could have been artifactual, for example, if inhibitory interneurons were more influenced by anesthesia. Additionally, baseline firing rates of the extracellularly recorded units were low, making detection of inhibition difficult (Aertsen and Gerstein, 1985). Finally, it is possible that cortical stimuli were not located optimally to evoke inhibition. Previous work suggests that inhibitory cortico-reticulospinal actions can be evoked from a narrow strip located at the anterior edge of M1 (Hines, 1943; McCulloch et al., 1946). The surface M1 electrodes in the current study were more posterior than this, which might explain the dominance of facilitatory effects.

### Resting membrane potential

The largest reported database of intracellular recordings from identified reticulospinal cells is from Chan and Chan (1983) in cat, who reported unusually low resting membrane potentials: between −12 and −40 mV for 83% of cells. Shimamura et al. (1980) also reported some cat reticulospinal cells with low resting potentials (range −10 to −65 mV). In the present recordings from primates, we found a wide range of resting potentials; half of the cells did not show resting potentials more negative than −40 mV. Under more stable *in vitro* conditions, Serafin et al. (1996) reported more typical resting membrane potentials in unidentified cells from the guinea pig nucleus gigantocellularis (mean −61 mV; cells were only accepted for study if potential was <−55 mV). Measurement of resting potential *in vivo* is often complicated by an incomplete seal between the membrane and electrode, and the precarious recording conditions. Our data are not incompatible with reticulospinal cells having typical resting potentials, with any discrepancies accounted for by such measurement artifacts. We equally cannot exclude that reticulospinal cells do have unusual membrane properties, but if so, this appears to be less marked in monkey than in cat.

### Conduction velocity of primate reticulospinal axons

Measurements of conduction velocity from intracellular recordings yielded a bimodal distribution, with peaks around 20 and 55 m/s (Fig. 10, top). By contrast, the faster peak was absent from the extracellular data, where velocities largely overlapped with the slower peak of intracellular measurements (Fig. 10, bottom). It is well known that extracellular recordings have a bias toward large cells with faster-conducting axons (Humphrey and Corrie, 1978; Kraskov et al., 2019, 2020). An even more extreme bias is likely for intracellular records, as penetrations into small cells are usually rapidly lost because of mechanical instability. An additional factor was the duration of the stimulus artifact, which was 0.65–1.55 ms for extracellular measurements, but only 0.44–0.7 ms for intracellular. This often prevented us from seeing the fastest antidromic responses in the extracellular measurements. The true distribution of conduction velocities is therefore likely to be a combination of the two datasets which we report.

Previous studies in cat reticular formation commonly found a bimodal conduction velocity, with a maximum from 100 to 150 m/s (Pilyavsky, 1975; He and Wu, 1985; Matsuyama and Drew, 2000). In monkey, epidural spinal volleys elicited by reticulospinal and corticospinal tract stimulation have similar latencies (Riddle et al., 2009), suggesting a similar maximum conduction velocity (measured for the monkey corticospinal tract as 60–94 m/s; Humphrey and Corrie, 1978; Firmin et al., 2014). This suggests that macaque reticulospinal fibers are slower than in cat, which is supported by the present direct recordings. In cat fast and slowly conducting reticulospinal cells may receive different inputs from the cortex. According to Pilyavsky (1975), fast RST cells receive only long-latency, whereas slow RST cells receive both early and long-latency corticoreticular inputs. By contrast, He and Wu (1985) found that fast RST cells had only short-latency, and slow RST cells only long-latency cortical inputs. The discrepancy in the literature is hitherto unexplained. In our data, fast monosynaptic inputs were common in both intracellular and extracellular recordings, suggesting that they occurred regardless of reticulospinal conduction velocity.

### Functional implications

Given the prevalence of motor cortical damage in humans, potential alternative routes through which movement can be controlled need urgently to be identified and understood. Plasticity in reticulospinal pathways following motor cortical damage is an obvious potential target (Zaaimi et al., 2012, 2018b; Darling et al., 2018; McPherson et al., 2018; Choudhury et al., 2019). Our results here however force us to conclude that cortico-reticulospinal outputs are configured to control movement bilaterally, and therefore play a very different role from the strongly lateralized corticospinal tract. Within one limb, we have previously shown that the reticular formation can effectively activate muscles, but not in the fractionated patterns used in flexible everyday movements (Zaaimi et al., 2018a). The reticular formation plays a more extensive role in gross, rather than fine, hand function (Lawrence and Kuypers, 1968; Baker and Perez, 2017; Tazoe and Perez, 2017), a point further emphasized by the convergence from different stimulation sites across M1 onto single reticulospinal neurons (Figs. 5, 9). It is possible that this distinction can be extended to a bilateral motor context: fine control of one limb is the preserve of the corticospinal tract originating from M1, whereas coordination of gross movements across two limbs may especially engage cortico-reticulospinal pathways, originating from PM as well as M1. Previous work suggested that SMA and M1 control bilateral postural adjustments during movement via a sub-cortical circuit (Massion et al., 1999). The cortico-reticulospinal connections described here would be ideal as the substrate for such a system, although we must modify past concepts slightly to encompass reticulospinal control of both distal and proximal muscles: corticoreticular outputs to RST cells were here more common from the lateral than medial part of M1, and our past work showed RST connections even to motoneurons projecting to the hand (Riddle et al., 2009). Patients who are recovering from damage to the motor cortex after a stroke frequently show involuntary mirror movements, which may have a sub-cortical origin (Ejaz et al., 2018). This may reflect an increased reliance on cortico-reticulospinal pathways with the attendant loss of fine, lateralized control.
